# Neurofibromatosis Type 2 Presenting with Oculomotor Ophthalmoplegia and Distal Myopathy

**DOI:** 10.1155/2016/1701509

**Published:** 2016-09-21

**Authors:** Jessica Mani Penny Tevaraj, Evelyn Tai Li Min, Raja Azmi Mohd Noor, Azhany Yaakub, Wan Hazabbah Wan Hitam

**Affiliations:** ^1^Department of Ophthalmology, School of Medical Sciences, Health Campus, Universiti Sains Malaysia, 16150 Kubang Kerian, Kelantan, Malaysia; ^2^Hospital Universiti Sains Malaysia, 16150 Kubang Kerian, Kelantan, Malaysia

## Abstract

Neurofibromatosis type 2 usually presents with bilateral acoustic schwannomas. We highlight the rare presentation of neurofibromatosis initially involving third nerve. A 23-year-old Malay female presented with left eye drooping of the upper lid and limitation of upward movement for 8 years. It was associated with right-sided body weakness, change in voice, and hearing disturbance in the right ear for the past 2 years. On examination, there was mild ptosis and limitation of movement superiorly in the left eye. Both eyes had posterior subcapsular cataract. Fundoscopy showed generalised optic disc swelling in both eyes. She also had palsy of the right vocal cord, as well as the third and eighth nerve. There was wasting of the distal muscles of her right hand, with right-sided decreased muscle power. Pedunculated cutaneous lesions were noted over her body and scalp. MRI revealed bilateral acoustic and trigeminal schwannomas with multiple extra-axial lesions and intradural extramedullary nodules. Patient was diagnosed with neurofibromatosis type 2 and planned for craniotomy and tumour debulking, but she declined treatment. Neurofibromatosis type 2 may uncommonly present with isolated ophthalmoplegia, so a thorough physical examination and a high index of suspicion are required to avoid missing this condition.

## 1. Introduction

Neurofibromatosis type 2 or central neurofibromatosis is an autosomal dominant disorder [1]. In more than 90% of patients, it presents with bilateral acoustic schwannomas or neurofibromas (BAN) [1]. We aim to report a rare case of neurofibromatosis type 2 which presented with right distal myopathy and left third nerve palsy six years prior to the development of acoustic changes. We also highlight relevant clinical examination and investigations which may assist in the diagnostic workup of this condition.

## 2. Case Report

A 23-year-old Malay female presented with drooping of the left eyelid and limitation of upward movement for 8 years. She had a history of poor vision in the right eye for the past 20 years. It was associated with progressive outward deviation of the right eye. She also had progressive right lower limb weakness that caused difficulty in walking. This was followed by involvement of the right upper limb. The weakness in both upper and lower limbs involved the distal muscles, followed by the proximal ones. She also noticed a change in voice and disturbance of hearing in the right ear for the past 2 years.

**Figure 1:**
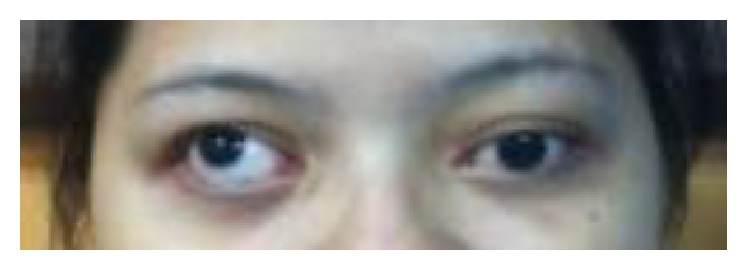
Patient presented with right lid retraction, exotropia, and hypertropia.

**Figure 2:**
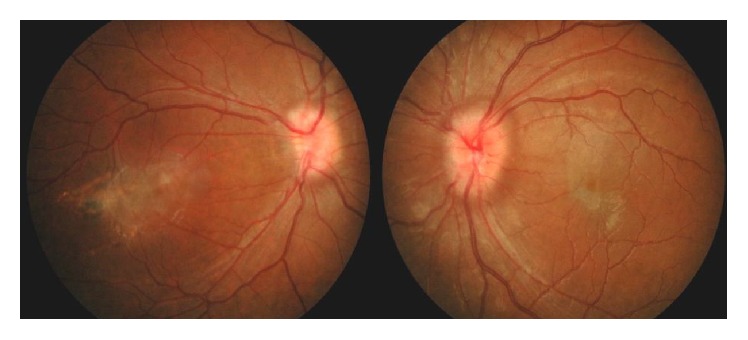
Bilateral generalised optic disc swelling with old right macular scar.

**Figure 3:**
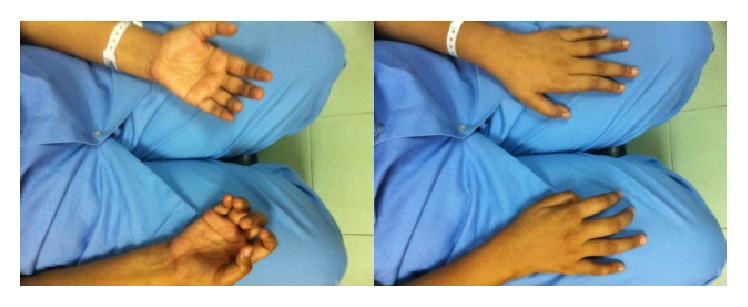
Muscle wasting of the right hand.

**Figure 4:**
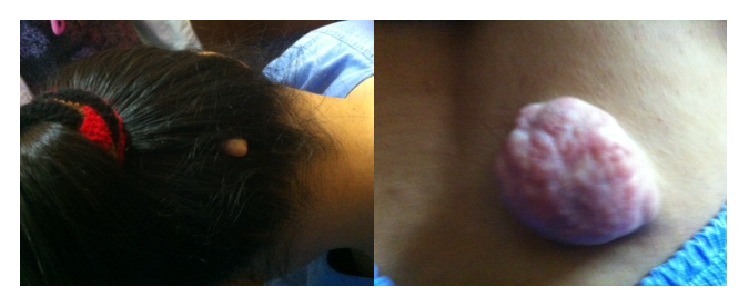
Mucocutaneous pedunculated neurofibromatous lesion on patient's scalp and lower back.

On examination, visual acuity was counting finger at 2 feet in the right eye, while in the left, it was 6/18, improving to 6/12 with pinhole. There was mild lid retraction in the right eye with exotropia and hypertropia, while the left eye had mild ptosis (Figure 1). The left eye demonstrated frontalis muscle overaction and limitation of movement superiorly. The pupils were normal. She had bilateral posterior subcapsular cataracts and generalised disc swelling, with slightly tortuous vessels. The right eye was amblyopic secondary to an old macular scar (Figure 2). On systemic examination, there was wasting of the distal muscles of her right hand, with decreased muscle power (Figure 3). A few pedunculated cutaneous lesions were noted over her body and scalp (Figure 4). Her right thigh had some café au lait spots. She also had sensory neural deafness and right vocal cord paralysis. The rest of the cranial nerves were intact.

**Figure 5:**
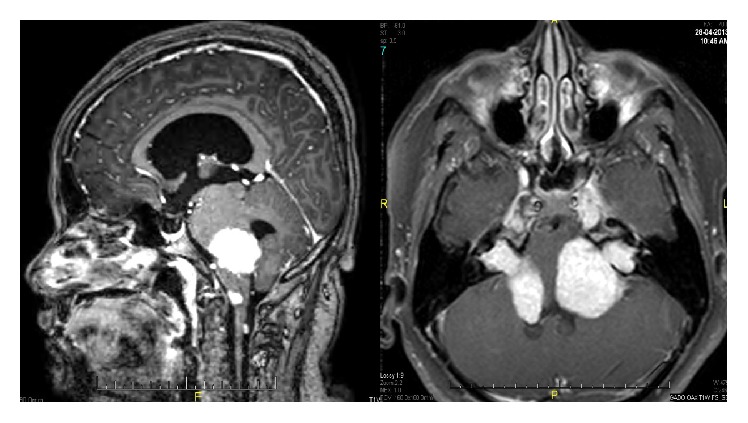
Brain MRI showing CP angle tumour, vestibular and trigeminal schwannoma, and bilateral concavity of cavernous sinuses.

MRI revealed bilateral acoustic and trigeminal schwannomas with multiple extra-axial lesions and intradural extramedullary nodules (Figure 5). There were also multiple intraspinal lesions with multiple peripheral nerve lesions, likely meningiomas or neurofibromas. Obstructive hydrocephalus causing tonsillar herniation was noted. Patient was diagnosed to have neurofibromatosis type 2 with skin, eye, and neural involvement. Patient was planned for craniotomy and tumour debulking by neurosurgical team.

## 3. Discussion

Neurofibromatosis type 2 is an autosomal dominant disease caused by mutation of chromosome 22 band q 11-13.1, called the NF2 gene (neurofibromin 2) or merlin gene (moesinezrin-radixin-like protein) [1]. It is a cytoskeleton gene with unknown function [2]. Despite the nature of inheritance of this gene, there are about 25% to 30% of cases with single occurrence within a family [3]. Neurofibromasis type 2 generally presents with auditory symptoms like hearing loss (35%), tinnitus (10%), and disequilibrium (8%) due to nervous system tumours like vestibular schwannoma, meningioma, glioma, and ependymoma [3]. Paediatric cases are generally diagnosed early by identification of skin-related tumours [4–7]. The most common ocular finding is subcapsular cataract [1], which was present in our patient.

Our patient presented initially with third nerve palsy and only developed auditory symptoms later. This is a very unusual presentation, as patients commonly present early with auditory symptoms [3]. It is of interest to note that ocular symptoms are more common in those belonging to a younger mean age group, compared to extraocular symptoms [8–10]. A study by Evans et al. stated that, in rare cases, mononeuropathy can occur, particularly in children, who generally present with facial palsy that partially recovers, squint (third nerve palsy) or hand or foot drop [11].

As in our patient, Egan et al. reported four cases of patients presenting with ptosis and superior rectus palsy [12]. This was postulated to be caused by a tumour of the ipsilateral third nerve typical of schwannoma or due to an expansive process in the temporal region close to the cavernous sinus causing neural ptosis. This is the likely cause of the third nerve palsy in our patient, as her MRI showed involvement of both cavernous sinuses. Due to the branching of the third nerve into superior and inferior divisions, a lesion around the anterior cavernous sinus or orbit may cause selective impairment of either division [13]. Impairment of function of the superior division would lead to ptosis and impairment of elevation of the eye [13].

Mass effect to the medulla or foramen magnum may compress its contents, including the glossopharyngeal, vagus, and spinal root of accessory nerve. However, in view of the selective and limited involvement of the superior laryngeal nerve in this case, hydrocephalus resulting in nerve compression is less likely. We postulate that, in this case, right vocal cord palsy may be due to a small schwannoma in the cervical, mediastinum, jugular, or foramen magnum, which may not have been obvious on imaging [14].

Peripheral schwannomas, meningiomas, and neurofibromas may cause weakness and numbness, which can progress to hand and foot drop. However, there were no previous studies documenting progressive myopathy, starting from the distal extremities and extending proximally. It is possible that this pattern of involvement occurred as patient progressed to the advanced stage of her disease.

As this is an autosomal dominant disease with significant morbidity, patients with family history of neurofibromatosis type 2 should be screened to detect the disease in the early stages, before irreversible damage occurs. Early presentation and intervention, before multiple cranial nerves are involved, will result in a better prognosis for this condition.

## 4. Conclusion

Although neurofibromatosis type 2 commonly presents with auditory symptoms due to development of an acoustic schwannoma, it may rarely present with distal myopathy or a third nerve palsy. Clinicians should have a high index of suspicion and perform a thorough systemic examination in these patients, with particular attention paid to dermatological changes. In view of the variable presentation of this disease, a multidisciplinary approach to screening should be instituted, and these patients managed under the combined expertise of the ophthalmology, otorhinolaryngology, neurology, and dermatology disciplines.
